# Obstetric fistulae in southern Mozambique: incidence, obstetric characteristics and treatment

**DOI:** 10.1186/s12978-017-0408-0

**Published:** 2017-11-10

**Authors:** Sibone Mocumbi, Claudia Hanson, Ulf Högberg, Helena Boene, Peter von Dadelszen, Anna Bergström, Khátia Munguambe, Esperança Sevene, the CLIP working group

**Affiliations:** 1 Department of Obstetrics and Gynaecology, Faculty of Medicine, Universidade Eduardo Mondlane (UEM), Av. Agostinho Neto 679, 1100 Maputo, Mozambique; 2 Department of Women’s and Children’s Health, Uppsala University, Akademiska sjukhuset, SE-75185 Uppsala, Sweden; 3 Department of Public Health Sciences, Karolinska Institutet, Tomtebodavagen 18A, Plan 4, Stockholm, Sweden; 4 Department of Disease Control, London School of Hygiene and Tropical Medicine, Keppel St, London, WC1E 7HT UK; 5 Centro de Investigação em Saúde de Manhiça (CISM), Rua 12, Vila da Manhiça, 1121 Manhiça, Mozambique; 6 Global Women’s Health, King’s College, London, UK; 7 University College London, Institute for Global Health, Gower St, London, WC1E 6BT UK; 8 Department of Public Health, Faculty of Medicine, UEM, Av. Salvador Allende 702 R/C, Maputo, Mozambique; 9 Department of Physiological Science, Clinical Pharmacology, Faculty of Medicine, UEM, Av. Salvador Allende 702 R/C, Maputo, Mozambique

**Keywords:** Obstetric fistula, Caesarean, Incidence, Population-based, Sub-Saharan Africa

## Abstract

**Background:**

Obstetric fistula is one of the most devastating consequences of unmet needs in obstetric services. Systematic reviews suggest that the pooled incidence of fistulae in community-based studies is 0.09 per 1000 recently pregnant women; however, as facility delivery is increasing, for the most part, in Africa, incidence of fistula should decrease. Few population-based studies on fistulae have been undertaken in Sub-Saharan Africa, including Mozambique. This study aimed to estimate the incidence of obstetric fistulae in recently delivered mothers, and to describe the clinical characteristics and care, as well as the outcome, after surgical repair.

**Methods:**

We selected women who had delivered up to 12 months before the start of the study (June, 1st 2016). They were part of a cohort of women of reproductive age (12–49 years), recruited from selected clusters in rural areas of Maputo and Gaza provinces, Southern Mozambique, who were participating in an intervention trial (the Community Level Interventions for Pre-eclampsia trial or CLIP trial). Case identification was completed by self-reported constant urine leakage and was confirmed by clinical assessment. Women who had confirmed obstetric fistulae were referred for surgical repair. Data were entered into a REDCap database and analysed using R software.

**Results:**

Five women with obstetric fistulae were detected among 4358 interviewed, giving an incidence of 1.1 per 1000 recently pregnant women (95% CI 2.16–0.14). All but one had Caesarean section and all of the babies died. Four were stillborn, and one died very soon after birth. All of the patients identified and reached the primary health facility in reasonable time. Delays occurred in the care: in diagnosis of obstructed labour, and in the decision to refer to the secondary or third-level hospital. All but one of the women were referred to surgical repair and the fistulae successfully closed.

**Conclusion:**

This population-based study reports a high incidence of obstetric fistulae in an area with high numbers of facility births. Few first and second delays in reaching care, but many third delays in receiving care, were identified. This raises concerns for quality of care.

**Electronic supplementary material:**

The online version of this article (10.1186/s12978-017-0408-0) contains supplementary material, which is available to authorized users.

## Plain English summary

Obstetric fistula is a childbirth complication in which a hole develops between the bladder and vagina or between the rectum and vagina. It occurs after a prolonged and neglected labour without adequate assistance or might happen as a complication after caesarean delivery, and causes a constant leaking of urine and/or faeces through the vagina.

There is no reliable data in Mozambique about the number of women suffering this preventable condition. This study was to estimate the number of women who developed obstetric fistula during the year before the start of the study (incidence), describe their clinical characteristics and the results of the surgical repair. Women with obstetric fistula were identified by self-reported constant urine leakage and the condition confirmed by physician’s examination.

Women who delivered in rural areas of Southern Mozambique, 12 months before June, 1st 2016, were interviewed about their delivery experiences, birth outcomes, complications and satisfaction with care. Among 4358 interviewed, five presented obstetric fistula, giving an incidence of 1 woman developing obstetric fistula for each 1000 recently pregnant women. The study shows that all women diagnosed with fistula, had arrived at the health centre in time. All but one had a caesarean delivery. Delays were identified in attendance, referral and care. Afterwards, all had successful fistulae’s surgical repair.

The conclusion was that the incidence of obstetric fistula is high in an area with a high facility-based delivery rate. We are concerned about the quality of care as many of the fistula cases occurred in the health facilities.

## Background

Obstetric fistula is one of the most devastating consequences of unmet needs in obstetric services. The World Health Organization (WHO) defines a fistula as an abnormal opening between the woman’s vagina and bladder and/or rectum, through which her urine and/or faeces continually leak [1]. Estimates indicate that two million women suffer from undetected or untreated fistulae globally, and that 50,000 to 100,000 new cases occur each year, mainly in low- and middle-income countries (LMICs) in sub-Saharan Africa and South Asia [2], where maternal mortality is high [3, 4]. While the most common cause of fistula is prolonged or obstructed labour [5, 6], recent studies suggest that an increasing proportion of urogenital fistulae in LMICs may be iatrogenic, mainly resulting from caesarean section [5].

Reported rates of obstetric fistula in LMICs vary widely, from 0 to 4.09 obstetric fistula cases per 1000 deliveries, and reliable data on the prevalence and incidence are sparse [7]. Most estimates are based on self-reporting, personal communication with surgeons, studies conducted by advocacy groups, and the review of hospital series. In all of these reports, the relevant denominators, such as the source population and number of births, are unknown or unreported [8–10]. Only six studies, all from LMICs, were judged to be of sufficient quality for a systemic review by Adler et al. (2013) [11]: three community-based studies [12–14]; and three hospital-based studies [15–17]. The pooled incidence of fistula ranged from 0.09 per 1000 recently pregnant women in the community-based studies to 0.66 per 1000 recently pregnant women in the hospital-based studies. The review highlighted that the included studies had important methodological limitations: most being retrieved population-based studies, which were not included in the estimate, and reported on self-reported occurrence of fistulae without physical examination by a trained healthcare provider. Alternatively, studies where obstetric fistulae were clinically confirmed, were primarily derived from hospitals, thus lacking a denominator and detailed information of the population from where the women originated.

Despite being more expensive, community-based surveys generally provide wider coverage, better representation of a regional or national population, and more opportunities to collect a wide range of data compared to facility-based studies [18]. However, without clinical examination, such studies cannot establish accurate disease rates, and making a correct diagnosis without eliminating other causes of incontinence is not possible [7, 19, 20].

Obtaining reliable epidemiologic data on fistula is important to efficiently direct programme efforts [1, 21]. In Mozambique there is an on-going programme for fistula prevention and treatment, which consists of raising awareness, early detection and surgery campaigns in all provinces, however, exact data on the prevalence and incidence are not available [22], thus limiting the effectiveness of programme planning [23]. In 2015, it was estimated that 100,000 women suffer from obstetric fistula in Mozambique [22], and only 556 were identified and underwent surgery for the condition during the campaigns for fistula treatment [Melo, A.; Unpublished data from the Mozambican National Programme for fistula prevention and treatment, 2015].

The objective of the current study was to describe the problem of obstetric fistulae in rural areas of Southern Mozambique. The specific aims were: to estimate the incidence of obstetric fistulae in recently delivered mothers, to describe the women’s obstetric profile and their self-reported health care, as well as to report outcomes after surgical repair.

## Methods

The study design was a survey within a population cohort. We interviewed recently delivered women (mothers), defined as those having given birth during the 12 months before the start of the study (June, 1st 2016). We did not, however, include those who had delivered in the two weeks preceding this time, as fistula usually occurs between one and 10 days after delivery. These mothers were identified within a cohort of women of reproductive age (12–49 years) from the ongoing Community Level Interventions for Pre-eclampsia (CLIP) trial (NCT01911494) [24], covering households in rural areas of Maputo and Gaza provinces, Southern Mozambique. The CLIP database was only used to facilitate the identification of the women and their households. We identified self-reported symptoms of fistulae and any suspected fistulae were confirmed by clinical assessment.

An obstetric fistula was defined as an abnormal opening between the woman’s vagina and bladder and/or rectum, through which her urine and/or faeces continually leak. The fistula incidence was calculated using the total number of women who delivered as the denominator.

### Study setting and participants

The study area included 12 rural clusters of Maputo and Gaza provinces in southern Mozambique, with an estimated population of 72,150 women of reproductive age and 17,400 births per year. The proportion of births attended by skilled health personnel in the study area is 85%, and the caesarean section rate is at 3.8% [Sacoor, C.; Unpublished data from CISM Demographic Surveillance, Manhiça, 2017].

The area is served by 32 health centres, providing essential preventive and curative services, which include basic antenatal care and assistance with normal births. Complicated cases are referred to the secondary level health facilities, which include four rural and one district hospital conducting routine surgical interventions, such as caesarean sections or obstetric hysterectomies, and which have larger diagnostic capacity. Two Provincial Hospitals provide tertiary-level care and one Central Hospital provides specialized care (quaternary level).

The sample size calculation was based on the prevalence of 0.15% reported by Adler et al. [11]A sample of 3700 births was deemed appropriate to estimate the prevalence with 0.05 significance level.

A list of 4441 recently pregnant women (mothers) was produced. Births (live and stillbirth) were approximated with equal numbers of pregnancies. We interviewed all mothers who we were able to recruit after a maximum of two attempts and who agreed to participate in the study.

Local authorities, including traditional leaders and village heads, were informed about the objectives of the study; a mobilization process that was important to ensure the participation of the mothers.

### Study instrument

The study instrument was part of a bigger study; an assessment of mothers’ experiences and birth outcomes. We used a questionnaire sequence aimed at women who had delivered a baby in the past year to collect information on their place of residence, place of birth, birth outcomes, childbirth complications, morbidity (including fistulae) and satisfaction with care, including incidences of disrespect and abuse during childbirth. Questions addressing fistulae were adapted from the United Nations Population Fund (UNFPA) proposal on obstetric fistula for the existing Demographic and Health Survey (DHS) fistula module questionnaire [25]. Information on birth outcomes was also retrieved from the antenatal and perinatal card (Caderneta de Saúde da Mulher). Mothers’ birth experiences and perceived health care responsiveness were measured by adapting selected questions from the World Health Organisation (WHO) Health System Responsiveness Questionnaire [26] and the Maikhanda study undertaken in Malawi [27, 28]. The questions were translated from English to Portuguese, and the questionnaire was pre-tested and piloted before application to ensure that the questions were clear and understandable. The questionnaire was programmed to be used on a tablet using ODK Collect version 1.4.6 [29, 30]. Clinical data from the mothers with suspected fistulae were collected using a standard form. An in-depth interview guide was used to collect the narratives of the mothers with confirmed fistula. The mothers’ questionnaire can be found in Additional file 1.

### Data collection

Data were collected by means of structured interviews at household level between June 1st, 2016 and October 28th, 2016 by 13 experienced female data collectors who had been trained on the protocol, the data collection forms, and the administration of informed consent. Special focus was placed on the appropriate approaches to take when asking sensitive questions and on when to communicate the Portuguese questions in the local language (Changana). Data collectors were monitored by the field supervisor to ensure their compliance with the study protocol. The supervisors performed random second interviews with 1% of the women to test the quality of the data and to determine whether the data collectors needed re-training. Once a week, the PI and the data management team reviewed both the completed questionnaire and the database to check for missing answers, duplications and inconsistencies, and, if needed, the data collector was sent back to the field to gather data where corrections and clarifications were necessary.

All interviewed mothers who responded ‘Yes’ to the question ‘Have you, AFTER your last pregnancy, ever experienced a constant leakage of urine or stool from your vagina during the day and night?’ were re-visited at home to be invited to participate and to schedule a day for clinical examination and appropriate diagnosis at the Manhiça District Hospital. A gynaecologist (SM) completed a standardised history and clinical examination. The diagnosis of fistula was confirmed by observation of urine leakage and clear visualization of the fistula. A dye test, with methylene blue, involved back-filling the bladder and temporarily occluding the urethra for evaluation of dye leakage. We used the Waaldijk classification [21, 31, 32]. Mothers with confirmed fistula were invited to provide an in-depth interview (IDI) to provide narratives of their experiences, which will be analysed and reported elsewhere. Information on the duration of labour was collected from the IDI and used for classification of first, second and third delays in birth attendance [33].

All mothers identified as having a fistula were referred to Maputo Central Hospital for surgical fistula repair. The study team provided transport and support throughout the process. Continence after surgical repair was assessed by asking about any involuntary urine leakage and by conducting a cough test.

### Data management and analysis

All data were electronically captured on-site and uploaded weekly to the Manhiça Health Research Center (CISM) database using REDCap version 6.14.0 (Vanderbilt University 2016) [34]. Database content was checked for missing answers, duplications and inconsistencies. Data were then exported to R software (version 3.3.1) for further analysis [35].

The incidence proportion, with 95% confidence interval, was determined by calculating the number of confirmed fistula cases as a proportion of the surveyed mothers who had given birth within 1 year prior to the interview. Descriptive statistics for each woman were obtained from the database.

## Results

### Incidence of fistula

**Fig. 1:**
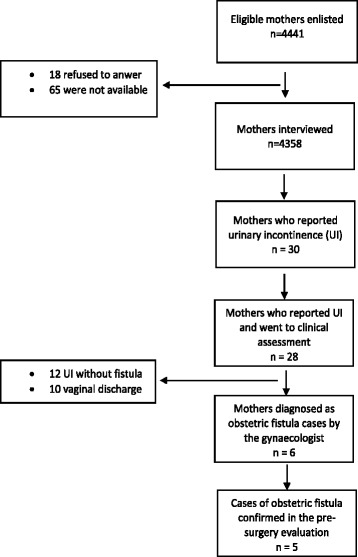
Diagram showing the flow through the study

Of all mothers registered as having delivered in the 12 months prior to the start of the study, 4358 were interviewed and only 18 did not agree to participate (Fig. 1). Out of all mothers, 10.4% delivered at home, 2.4% delivered on the road to a health facility, and 87.2% delivered in facilities. Of the facility deliveries, 0.5% had ventouse, and 3.6% had caesarean section.

Out of the 30 mothers who reported a constant leakage of urine, two did not agree to be examined, and 28 went on to attend at the Manhiça District Hospital for clinical examination by the gynaecologist. Six mothers were diagnosed as having obstetric fistula and were referred for surgical repair. The pre-surgery examination confirmed obstetric fistula (all vesico-vaginal) in 5 mothers. The sixth mother had severe urinary incontinence, misdiagnosed as intra-cervical fistula. From the remaining examined mothers, urinary incontinence was confirmed in twelve cases, and ten were in fact vaginal discharge without urinary incontinence. Thus, in total, five cases of vesico-vaginal fistulae were diagnosed out of 4358 mothers interviewed. We estimated the fistulae incidence among recently pregnant women to be at 1.1 per 1000 recently pregnant women (95% CI 2.16–0.14).

During the study’s implementation, a further nine women came voluntarily to the study hospital seeking care for fistula-like symptoms. These were observed and a diagnosis of fistula confirmed, but the women were not included in the study because the fistula happened more than one year before the study began. All were referred to the Central Hospital for surgery.

### Obstetric and clinical characteristics of the women affected by fistula

The characteristics of the five mothers diagnosed with recent obstetric fistulae are given in Table 1. The median age was 21 years, and four were aged under 24 years. Three were primiparas. Two had been identified has having a height of less than 150 cm during the antenatal consultations, but did not receive special attention regarding this obstetric risk. All five reported seronegative HIV status.

**Table 1:** Background, perinatal characteristics and outcome of the five mothers diagnosed with obstetric fistula in Maputo and Gaza provinces, Mozambique 2016

Characteristics	Fistula (*n* = 5)
Age (years)	
15–19	2
20–29	3
Education	
No education	1
Primary	4
Parity	
One birth	3
Two to fourth births	2
Previous stillbirth	
Yes	0
No	5
Previous neonatal death	
Yes	1
No	4
Mode of delivery	
Vaginal	1
Caesarean	4
Perinatal outcome	
Stillbirth	4
Neonatal death	1

The fistula characteristics are presented in Table 2. Three were type I fistula; the other two were: types II A and B. An assessment of the probable mechanism of the fistula (ischaemic, iatrogenic or combination) is presented for each participant.

**Table 2:** Labour, outcome, fistula characteristics and repair of the 5 mothers diagnosed with vesico-vaginal fistulae in Maputo and Gaza provinces, Mozambique 2016

Case	Age	Obstetric history	Time taken to				Labour duration	Type of delivery (at the secondary HF)	Fistula description and type (Waaldijk classification)	Assessment of the mechanism of the fistula	Result of surgical repair	Follow up 3 months after the surgery
			Seek care, from the first pain (Phase I delay)	Identify and reach the primary HF (Phase II delay)	Receive treatment until transfer to the secondary HF (Phase III delay)	Receive treatment at the secondary HF (Phase III delay)						
1.DJC	19	G1P1	7 h	6 h	±24h^a^	No delay	>24 h	C/S	1 cm, mid-vaginal, non-circumferential involving the proximal urethra (bladder neck) with mild fibrosis (Type IIAa)^d^	Ischaemic	Closed	Closed and continent
2.FBM	27	G4P4	1 h	2 h	±16 h	No delay	<24 h	C/S	1 cm, “high” close to the cervix, intact urethra, mild fibrosis (Type I)^e^	Iatrogenic?	Not submitted to surgery*	N/A
3.PJM	22	G2P2	Went to maternity waiting home with 8 months	DK	±12 h^b^	±6 h	>24 h	Vaginal	Mid-vaginal, 1 cm, intact urethra. No fibrosis. (Type I)^e^	Ischaemic	Closed	Closed and continent
4.AJZ	18	G1P1	6 h	2 h	±12 h	±10 h	>24 h	C/S	Punctate 0.5 cm, “high” vaginal, with mild fibrosis. Intact urethra (Type I)^e^	Combination ischaemic and iatrogenic?	Closed	Did not come for follow up
5.CCM	21	G1P1	7 h	3 h	±24h^c^	DK	>24 h	C/S	large calibre, with total urethral damage (Type IIBb)^f^	Ischaemic	Closed	Closed, but still incontinent

*G* gravidity, gravida, *P* parity, para, *DK* don’t know, don’t remember

^a^went to the health centre (maternity) reporting pain, but stayed 6 days before starting to have effective contractions, ^b^stayed 2 weeks at the maternity waiting home before labour started. Had delay during transfer: arrived at the district hospital she had been sent to the provincial hospital, ^c^ went to the health centre (maternity) reporting pain, but stayed 4 days there before the labour started. Said she lost consciousness during labour (probably eclampsia?)

^d^Type IIAa: fistula involving the proximal urethra without circumferential defect, ^e^Type I: fistula with intact urethra, ^f^Type IIBb: fistula with total urethral damage and circumferential defect, *Receiving treatment for psychiatric complication

### Patient-reported experiences of labour

The mothers’ decision to seek care was made in a timely manner, between a minimum of 1 h and a maximum of 7 h. All used a private car or taxi to reach the primary health care facility and took no more than 6 h to arrive. Regarding the deliveries (Table 2), all reported having been referred from the primary health care facility to the secondary hospital. Labour duration, estimated from the first referred pain, ranged between 12 to 48 h and ended in caesarean section in four cases. The mothers reported having experienced delays at the health facilities, particularly in making the decision to perform operative delivery. One mother stayed for 2 weeks at the maternity waiting home and the day the labour started it took 12 h before she was referred to the hospital. Two mothers arrived at the primary health facility with minor pain and one stayed for 4 days and the other 1 week, before the decision to transfer was made. The mother who stayed for 1 week was told at the moment of the transfer that the baby was not alive and was in pelvic presentation. An ambulance was used to transfer four mothers to the next level of care, while one family had to hire a taxi.

During labour, all women were supported by a nurse and the caesarean sections were performed by non-physician clinicians [36]. All the caesareans but one were performed at the same district hospital in Gaza Province.

All of the babies died; four were stillborn, and one died very soon after birth. All mothers rated their previous labour as being very difficult. None of them felt they had been humiliated or treated disrespectfully and no one reported any physical abuse. All four who had had a caesarean section felt that the intervention was necessary.

### Fistula onset and treatment

Four mothers noticed the onset of the urine leakage one-to-two days after the caesarean section was performed; the only patient who had had a vaginal delivery noticed it 5 days after. The complications were recognized at the hospital, but no surgical treatment was proposed to them. All agreed that suffering a fistula is a big problem and none of the mothers were aware that fistula is treatable. Their overall health was perceived as good, except for one mother, who said it was neither good nor bad. Only one of the two, who referred to the abdominal pain as “terrible”, had received any treatment. None of the mothers had had intercourse since the appearance of the fistula.

During this study, fistula repair was offered to all of the mothers diagnosed with fistula. One mother, however, had a contraindication for surgery (receiving treatment for psychiatric complications) and was hence not referred for surgical repair. All of the remaining four fistulae were successfully closed. Of the three mothers who came for follow-up, one reported urinary incontinence, but there was no breakdown in the surgical repair.

## Discussion

### Main findings

Our findings indicate a community-based incidence of obstetric fistula of 1.1 per 1000 recently pregnant women. Caesarean section (CS) was performed for all but one of the deliveries causing the fistulae. Distance and access to the health care facility does not seem to be a reason for the delay in receiving the emergency caesarean section. All of the mothers identified and reached the primary health facility in reasonable time. A Type 3 phase of delay occurred in the management of these cases: delay in diagnosing obstructed labour, deciding the need of a CS, and the transferring of the patient to a higher level hospital. All but one woman with fistulae were referred for surgical repair and the fistulae were successfully closed. Incontinence persisted in one case after 3 months.

### Strengths and limitations

The major strength of our study is the community-based identification of the suspected cases (with reported symptoms) combined with hospital-based clinical examination, which maximised the likelihood of accurate case identification. The population of recently pregnant women was identified from a well-identified cohort of pregnant women and was followed over a defined period of time, minimising the risk of selection bias. Detection bias was minimized by the use of a standardized data collection instrument (fistula module) together with clinical examination.

Although nondisclosure of incontinence symptoms in the questionnaire interview was possible, it seems unlikely, given that the data collectors were trained to explain the meaning of urinary incontinence and to explain that treatment would be offered free of charge to those detected and to emphasise that all the information collected would be kept confidential. A risk of underestimating the number of fistula cases should be considered, as two of the 30 mothers who reported incontinence symptoms did not attend for subsequent examination. The ostracism and stigma associated with the condition [37] might have deterred the interviewed mothers from disclosing their symptoms due to the risk of being excluded from their households and/or communities. A further limitation was that we had no information about the pre- or post-operative circumstances.

### Interpretation

The fistula incidence of 1.1 per 1000 recently pregnant women that we found is higher than Adler’s [11] pooled incidence estimates in LMICs of 0.09 and 0.66 per 1000 recently pregnant women in community-based and hospital-based studies, respectively. It is recognized in the literature that reported rates of obstetric fistula vary widely. Taking into account that our survey was followed by gold-standard gynaecological exams, the variation could represent true differences in incidence. We emphasize the importance of the clinical examination and confirmation of all women who reported fistula symptoms: 78% of the mothers who reported fistula-like symptoms had conditions other than fistula. Similar to our study, a community-based screening for fistulae in Nigeria, using the fistula module questionnaire, found that 53% of the women who reported symptoms did not have a fistula [38].

Our estimate is close to the fistula incidence of 1.2 per 1000 births found in the only prospective population-based study, which was undertaken in rural West Africa in 1999 [12]. That incidence was, similar to our study, observed in rural areas where women are at higher risk of labour complications. However, the study from West Africa was undertaken in an area with a much lower proportion of births attended by skilled health personnel (39.6% vs 85%) and lower caesarean section rates (0.7% vs 3.8%). Although the low caesarean section rate could suggests an unmet need [39, 40], it is important to consider that most of the identified cases in this study occurred amongst women who arrived in a timely manner at the primary health facility (except one who took 13 h). Thus, while several studies have described delays in deciding to seek care (first delay) and accessing a health facility (second delays) as being important factors in fistula formation [41–43], we found most delays occurring at health facilities (third delay). Indeed, the detection of high-risk deliveries and decision to refer women to a hospital with surgery capability was not timely. In addition, delays in reaching the referral hospital, mostly due to unavailability of ambulances, have been observed, similar to those described by Waiswa et al. (2017) [44] from Southern Tanzania, where there are no ambulances at the primary facility level, which is why emergency transport is a constraint. These findings that the third delay (receiving inadequate care at health facility) contributed to obstetric problems are corroborated by other studies conducted in Tanzania, Gambia and elsewhere [45, 46]. We have classified the failure to detect obstetric problems in a timely manner at the primary health facility as a third delay, according to the model described by Thaddeus and Maine (1994) [33] and Berhan and Berhan (2014) [47]. However, as discussed by Gabrysh and Campbell (2009) [48], these delays can also be classified as second emergency delays, when complications that cannot be managed at the primary facility require referral to a higher-level facility. Whatever the classification, we consider that the issue is the capacity of the health system in caring for the women in labour.

Prolonged obstructed labour is the most common cause of fistula formation [2, 49, 50]. Three of the five identified fistulae were probably ischaemic in origin (two type II fistula and one type I). However, in two cases, the type I fistulae were high vaginal, close to the cervix. This type of fistula (equivalent to type 1 on the Goh classification [51], i.e., more than 3.5 cm from the external urinary meatus) is presumed to be iatrogenic in aetiology [5]. This could be the case for the type I fistula, which occurred where the duration of labour was less than 24 h. A combination of ischaemic and iatrogenic mechanisms could be suggested for the other type I, where the duration of labour was 30 h. It is important to mention that, for a type I fistula to be considered as iatrogenic, it would either mean that the incision of the uterus was too extensive or that the sutures used for the closure of the uterus would have included the fundus or posterior wall of the bladder and would have to be extended to the vesico vaginal septum. Although the mechanism involved could not be verified in this study, there are reports of an increasing proportion of iatrogenic urogenital fistulae in LMICs, primarily in obstetric interventions, especially caesarean section [5, 52], and it is possible that this was the case in our study. This calls for more quality of care research on obstructed labour and caesarean delivery.

All four foetuses delivered by caesarean section were stillborn. We do not have information about the circumstances of the management of these cases, but, considering that some of these could have been obstructed labour with foetal death, we should consider whether craniotomy may have been an alternative method of delivering these babies. This procedure is performed in LMICs [53–55] and is especially useful in patients who come from rural areas and run the risk of the complications of an abdominal-route delivery and the risks of rupture of uterine scar during subsequent pregnancies or labour [56, 57].

Although the fistulae were recognized at the health facilities, no treatment was proposed to these mothers at the time. This could be due to the limited availability of fistulae repair services and a lack of fistulae surgeons, which was reported in several settings in sub-Saharan Africa, including Mozambique, where at least 80% of women with fistula are estimated to have no access to fistulae repair each year [58]. However, the lack of adequate postpartum assessment and referral of the women with fistulae should be also considered.

This is the first study in Mozambique estimating fistula incidence in rural areas through a community-based survey with suspected obstetric fistulae being examined and treated at a health facility. Our results could be an underestimate for the country’s epidemiology as a whole, considering that our study area has a high density of health facilities compared to other parts of the country where more first and second delays would be expected.

## Conclusion

This population-based study reports a high incidence of obstetric fistulae in an area with a high rate of facility births. The most frequent delay was that of receiving care once at a health facility as opposed to delays relating to deciding to seek care or reaching the health facility. This raises concerns regarding the quality of care provided to women during delivery. Furthermore, our results suggest the need to improve postpartum care, especially for early identification and adequate management of obstetric fistulae. Future studies, particularly those that focus on quality of care in obstructed labour, use of caesarean delivery and role of alternative options such as craniotomy in cases of foetal death to avoid iatrogenic fistula, should be considered.

## Additional file

Additional file 1: Health survey with women having delivered during the last year. (PDF 835 kb)

## Abbreviations

CISM: Manhiça Health Research Center
CLIP: Community Level Interventions for Pre-eclampsia
CS: Caesarean section
DHS: Demographic and Health Survey
IDI: In-Depth Interview
LMIC: Low and Middle Income countries
UEM: Eduardo Mondlane University
UNFPA: United Nation Population Fund
WHO: World Health Organisation
